# Comprehensive Analysis of RNA-Seq in Endometriosis Reveals Competing Endogenous RNA Network Composed of circRNA, lncRNA and mRNA

**DOI:** 10.3389/fgene.2022.828238

**Published:** 2022-03-22

**Authors:** Meichen Yin, Lingyun Zhai, Jianzhang Wang, Qin Yu, Tiantian Li, Xinxin Xu, Xinyue Guo, Xinqi Mao, Jianwei Zhou, Xinmei Zhang

**Affiliations:** ^1^ Department of Obstetrics and Gynecology, Women’s Hospital, School of Medicine, Zhejiang University, Hangzhou, China; ^2^ Department of Gynecology, The Second Affiliated Hospital of Zhejiang University School of Medicine, Hangzhou, China

**Keywords:** endometriosis, non coding RNAs, competing endogenous RNA, circular RNA, long non coding RNA

## Abstract

Although long non coding RNAs (lncRNAs) and circular RNAs (circRNAs) play important roles in the pathogenesis of diseases, endometriosis related lncRNAs and circRNAs are still rarely reported. This study focused on the potential molecular mechanism of endometriosis related competitive endogenous RNA (ceRNA) composed of lncRNAs and circRNAs. We performed high-throughout sequencing of six normal endometria, six eutopic endometria and six ectopic endometria for the first time to describe and analyze the expression profile of lncRNA, circRNA and mRNA. Our results showed that 140 lncRNAs, 107 circRNAs and 1,206 mRNAs were differentially expressed in the ectopic group, compared with the normal and eutopic groups. We established an lncRNA/circRNA-mRNA co-expression network using pearson correlation test. Meanwhile, the results of Gene set enrichment analysis analysis showed that the 569 up-regulated differentially expressed mRNA (DEmRNA) were mainly related to the epithelial-mesenchymal transition, regulation of immune system process and immune effector process. Subsequently, we established a DElncRNA-miRNA and DEcircRNA-miRNA network using the starbase database, identified the common miRNAs and constructed DElncRNA/DEcircRNA-miRNA pairs. miRDB, Targetscan, miRwalk and circRNA/lncRNA-mRNA pairs jointly determined the miRNA-mRNA portion of the circRNA/lncRNA-miRNA-mRNA co-expression network. RT-qPCR results of 15 control samples and 25 ectopic samples confirmed that circGLIS2, circFN1, LINC02381, IGFL2-AS1, CD84, LYPD1 and FAM163A were significantly overexpressed in ectopic tissues. In conclusion, this is the first study to illustrate ceRNA composed of differentially expressed circRNA, lncRNA and mRNA in endometriosis. We also found that lncRNA and circRNA exerted a pivotal function on the pathogenesis of endometriosis, which can provide new insights for further exploring the pathogenesis of endometriosis and identifying new targets.

## Introduction

Endometriosis (EMS), the presence of extrauterine endometrial glands and stroma, is a common chronic disease affecting 10% of women in reproductive age. It is also the leading cause of subfertility or infertility in premenopausal women. However, its precise prevalence in the population is difficult to determine because it is asymptomatic or subclinical in most cases (El-Toukhy, 2020). Numerous hypotheses of EMS exist, including: embryonic stem cell origin, retrograde menstruation, implantation or coelomic metaplasia. Evidence accrued in recent years indicates that changes in the immune response can contribute to EMs. As we all know, endometriosis is an inflammatory disease. One pathogenesis of EMS is alterations in cell-mediated and humoral immunity. Immune responses are not only involved in the early immune escape of endometriotic cells, but also enhance the establishment and growth of endometriotic lesions (Zhang et al., 2018). Although in-depth and extensive studies have taken place, the specific pathogenesis of EMS has not been clarified. In terms of treatment of EMs, despite several available treatment options to relive, no real cure exists.

The recent application of next-generation sequencing has revealed thousands of non coding RNAs(ncRNAs). ncRNAs are functional RNAs transcribed from DNA, but not translated into proteins(Nothnick et al., 2015). The human genome project found that about 80% of human DNA is transcribed into RNA, of which only 2% of messenger RNA (mRNA) is translated into proteins, and most of the remainder is called non-coding RNA, including miRNA, lncRNA, circRNA. ncRNAs are important regulators of cell function and are widely involved in a variety of disease processes. Functional experiments show that ncRNAs are an important part of the pathogenesis of endometriosis and are related to the genetic risk associated with endometriosis (Sapkota et al., 2017). Moreover, lncRNAs regulate the expression of protein coding genes through cis acting on adjacent genes and trans on distal genes. Therefore, the expression level of lncRNAs is related to the expression level of the target gene mRNA (Liao et al., 2011).

Currently, little is known about the effect of lncRNA and circRNA in EMS. Therefore, it is necessary to comprehensively analyze lncRNA and circRNA to explore the function of lncRNA/circRNA-miRNA-mRNA ceRNA network in EMS. In this study, we analyzed the expression profiles of lncRNA, circRNA and mRNA using RNA sequencing and predicted the related functions of upregulated DEmRNA by GSEA. The results of GSEA indicated that DEmRNA were mainly enriched in epithelial-mesenchymal transition, positive regulation of immune system process and immune effector process. Subsequently, we established the circRNA/lncRNA-miRNA-mRNA ceRNA network based on database prediction and expression correlation analysis. The RT-qPCR results of clinical samples confirmed that the expression trend and correlation of important molecules in the co-expression network were consistent with the hypothesis.

This is the first study to construct circRNA/lncRNA-miRNA-mRNA co-expression network by analyzing DElncRNA, DEcircRNA and DEmRNA from sequencing data in EMS. Our findings may lead to the discovery of a new pathogenesis of EMS and offer new theories for treatment.

## Methods

### Sample Collection and RNA Extraction

This research proposal was approved by the Women’s Hospital of Zhejiang University School of Medicine. In accordance with the Declaration of Helsinki, all patients received written informed consent prior to enrollment. Six paired ectopic endometrium and eutopic endometrium samples were selected and prepared for RNA sequencing from six patients with ovarian EMs cysts diagnosed by laparoscopic surgery. Meanwhile, six normal endometrial samples were obtained from six patients who underwent hysteroscopy for endometrial polyps.

Fresh tissue specimens were immediately frozen in liquid nitrogen and stored at −80°C, Samples were sent to Genenergy Biotechnology (http://www.genenergy.cn/) for RNA extraction. According to the manufacturer’s instructions, a sequencing bank was created using the TruSeq RNA Sample Preparation Kit (Illumina). Illumina HiSeq X Ten was used to analyze 151-BP paired sequences of the library.

### Identification of DERNAs

In order to identify the differentially expressed lncRNA and mRNA in ectopic endometrium, eutopic endometrium and normal endometrium, differential expression profiles were analyzed. DElncRNA and DEmRNA were distinguished according to |log2 (fold change)| > 3.0 and FDR <0.0001. DEcircRNA and were distinguished according to |log2 (fold change)| > 1 and *p* value <0.05. According to the above criteria, DERNA was screened from the expression profiles of the ectopic endometrium vs. normal endometrium and ectopic endometrium vs. eutopic endometrium respectively, and the common part of the two was regarded as the DERNA of this study. Venn diagrams were used to visualize the share DERNA between the two datasets for further analysis.

### GSEA

Gene set enrichment analysis (GSEA) was performed to identify significantly enriched groups of DEmRNA (Subramanian et al., 2005). In this study, GSEA software was applied to analyze biological pathway divergences, KEGG and hallmarks between ectopic and non-ectopic samples. *p* < 0.05 was considered the threshold value for statistical significance.

### Establishment of DEcirc/lncRNA-DEmRNA

The interaction between DEmRNA and DElncRNA was identified by the lncRNA-mRNA co-expression network. The basis of this construct is the normalized signal intensities of specific expression levels of mRNAs and lncRNAs. To construct the LncRNA-mRNA co-expression network, pearson correlation analysis was used to calculate statistically significant associations. The lncRNA-mRNA pairs with a rho ≥0.6 and *p*＜0.05 were selected, as these parameters indicated that the lncRNA-mRNA pairs were significantly co-expressed. Next, we conducted pearson correlation analysis between DEmRNA and DEcircRNA, and these DEmRNA were newly obtained DEmRNA related to DElncRNA expression (|rho|≥0.6, *p*＜0.05). DEcircRNA/DElncRNA-mRNA interactions were mapped using Cytoscape software (3.8.2) (Janet et al., 2021).

### DEcircRNA/lncRNA-miRNA

According to the ceRNA hypothesis, matching circRNA/lncRNA, miRNA and mRNA is crucial. Thus, this network may highlight a new molecular mechanism involved in the development of endometriosis. Pairs of miRNA-DElncRNA were established using the starbase database(PaciP et al., 2014; Li et al., 2014)

DEcircRNA-miRNA was constructed with miRanda software. The miRanda algorithm comprehensively predicts miRNA target genes through two steps of miRNA-circRNA sequence matching and energy stability evaluation. The algorithm uses a dynamic programming algorithm to search the region where the sequences of miRNA and circRNA are complementary and stable to form double strands. Threshold parameters used in predicting miRNA target genes are: S > 150, ΔG<−20 kcal/mol and Demand strict 5′ seed pairing, where S refers to single-legs-pair match scores in the matching area; ΔG is the free energy of double strand formation that constructs a global network of miRNA and target circRNA. The final DEcircRNA-miRNA pairs were the common part of the top 50 DEcircRNA-miRNA in normal vs. ectopic datasets and the top 50 DEcircRNA-miRNA in eutopic vs. ectopic datasets.

### Construction of lncRNA/circRNA-miRNA-mRNA Network

A circRNA/lncRNA–miRNA–mRNA ceRNA network was constructed based on the targeted relationships. Three miRNAs in this network were the intersection of predicted miRNA in both lncRNA-miRNA and circRNA-miRNA pairs. The target mRNAs of these three miRNAs were obtained from the TargetScan, miRDB, and miRwalk2.0 databases (Wang, 2008; AgarwalV et al., 2015; Dweep and Gretz, 2015). In order to improve the reliability of the results, we only selected those miRNA-mRNA relationship pairs that overlapped in all three databases for further research. Furthermore, the mRNAs predicted in the three data sets do not all belong to our ceRNA network, only mRNA closely related to the expression of lncRNA and circRNA could be considered as mRNA in the ceRNA network.

### Clinical Specimen Collection

This study included patients who had surgical treatments at Zhejiang University’s Women’s Hospital’s Department of Gynaecology during October and November of 2021. The endometrial samples were taken from women who had regular menstrual cycles and had not been treated with steroid hormones in the previous 3 months. Controls (*n* = 15) were endometrial samples from patients without endometriosis, adenomyosis, or other malignant illnesses. Laparoscopy and histological examination of patients with endometriosis (ectopic, *n* = 25) were used to confirm the diagnosis.

### RT-qPCR

The results of RNA-seq need to be verified by RT-qPCR. Trizol was used to extract the total RNA in the endometrial tissues of the control group and ectopic group. The concentration and purity of RNA were evaluated using a Nanodrop spectrophotometer (Thermo Fisher, United States). When the 260/280 ratio of RNA was >1.8, it was reverse transcribed to cDNA using the PrimeScriptTM RT reagent Kit with gDNA Eraser (Takara, Japan). The reverse transcription products were amplified using the Applied Biosystems ViiA™ seven system (ABI, United States) with the SYBR^®^ Premix Ex TaqTM kit 8 (Takara, Japan). Specific primers were synthesized by Generay (Shanghai, China), and the sequences are presented in Table 1. The relative expression was normalized by GAPDH, and the 2-ΔΔCt method was used to calculate the relative expression (Liu et al., 2020).

**TABLE 1:** Primer sequences for quantitative real-time polymerase chain reaction.

Name	Primer type	Primer sequence
circGLIS2	Forward	CAG​CAG​CTC​GCT​GTC​CCC​CGA​GCG
circGLIS2	Reverse	GTT​GGA​GGT​GGC​AGC​AGG​CAG​TGG
circFN1	Forward	GGA​GAA​GTA​TGT​GCA​TGG​TGT​CA
circFN1	Reverse	TGC​AGA​TTT​CCT​CGT​GGG​TTG
LINC02381	Forward	CCC​TGC​CCA​TAA​GCT​ACT​CA
LINC02381	Reverse	AAC​TTT​GAC​CCC​CAA​ATG​CC
IGFL2-AS1	Forward	AGT​TCC​TGA​TTT​CAG​CCC​CA
IGFL2-AS1	Reverse	TCC​TGG​GTT​GAC​AGG​GTA​GAA
CD84	Forward	GGA​GAA​GAG​GGT​AAT​GTC​CTT​CA
CD84	Reverse	CCA​TTG​CGA​TGT​CTG​CAC​A
LYPD1	Forward	GGC​AAC​TTT​TTG​CGG​ATT​GTT
LYPD1	Reverse	CGT​TCA​CCG​TGC​AAT​TCA​CA
FAM163A	Forward	ATG​ACA​GCG​GGA​ACG​GTT​G
FAM163A	Reverse	GTA​GCA​CAG​GAC​GGC​AAT​GAT

### Statistical Analysis

Statistical analyses were performed by the GraphPad Prism 7 (GraphPad, CA, United States) and SPSS 22.0 software packages (SPSS, IL, United States). Statistically significant differences between groups were estimated by Independent-Sample *t* test. The results were evaluated using Spearman’s correlation coefficient test. All values are expressed as the mean ± standard error of the mean; *p* < 0.05 was considered statistically significant.

## Results

### Differential Expression Profile of mRNAs, lncRNAs and circRNAs

**FIGURE 1:**
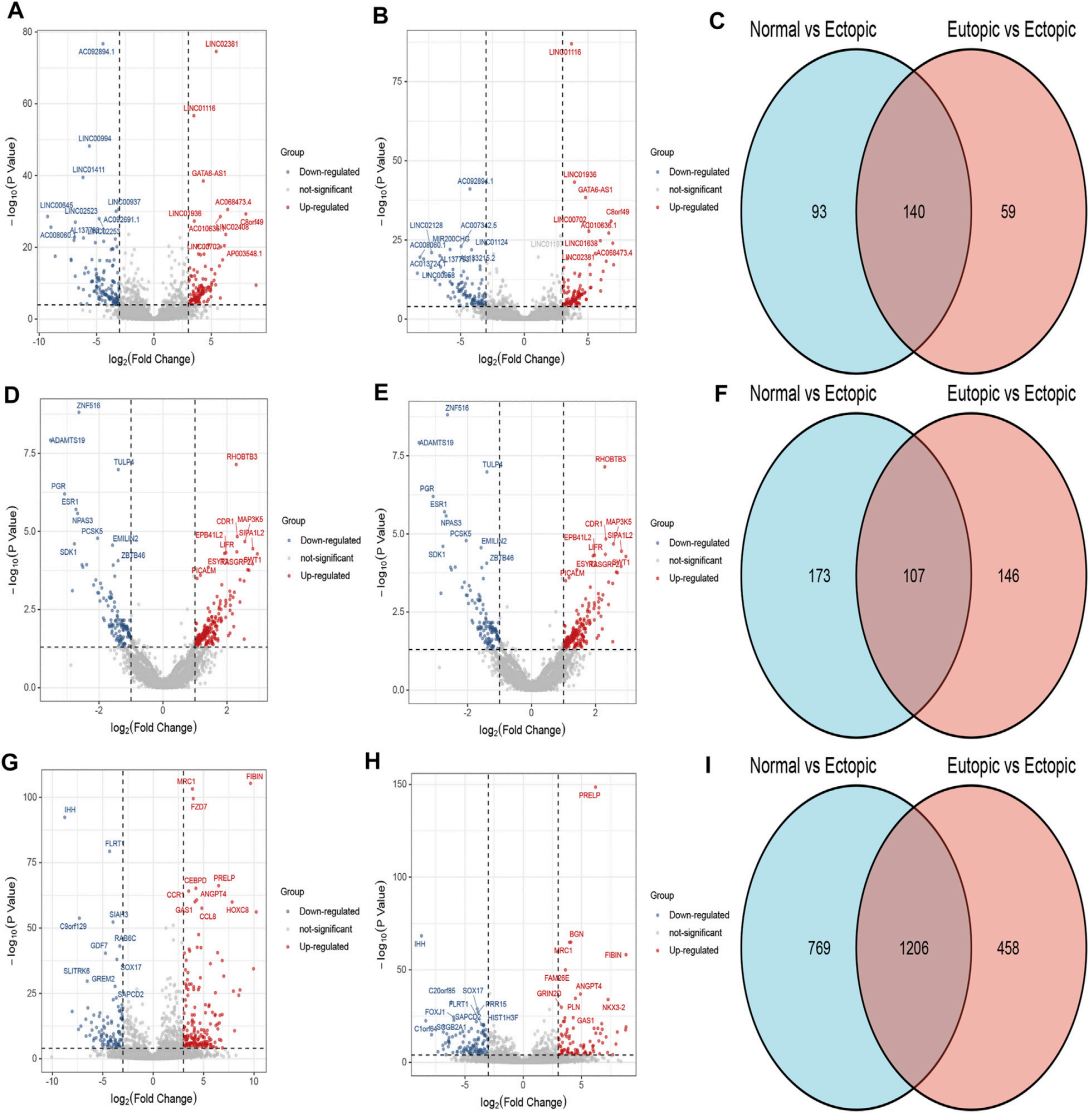
DElncRNA, DEcircRNA and DEmRNA in normal endometrium vs. ectopic endometrium and eutopic endometrium vs. ectopic endometrium datasets. **(A)** Volcano plot of DElncRNA in normal endometrium vs. ectopic endometrium dataset. **(B)** Volcano plot of DElncRNA in eutopic endometrium vs. ectopic endometrium dataset. **(C)** Venn diagram of common part of DElncRNA in normal endometrium vs ectopic endometrium and eutopic endometrium vs. ectopic endometrium datasets. **(D)** Volcano plot of DEcircRNA in normal endometrium vs. ectopic endometrium dataset. **(E)** Volcano plot of DEcircRNA in eutopic endometrium vs. ectopic endometrium. **(F)** Venn diagram of common part of DEcircRNA in normal endometrium vs ectopic endometrium and eutopic endometrium vs. ectopic endometrium datasets. **(G)** Volcano plot of DEmRNA in normal endometrium vs. ectopic endometrium dataset. **(H)** Volcano plot of DEmRNA in eutopic endometrium vs. ectopic endometrium dataset. **(I)** Venn diagram of common part of DEmRNA in normal endometrium vs ectopic endometrium and eutopic endometrium vs. ectopic endometrium datasets.

**FIGURE 2:**
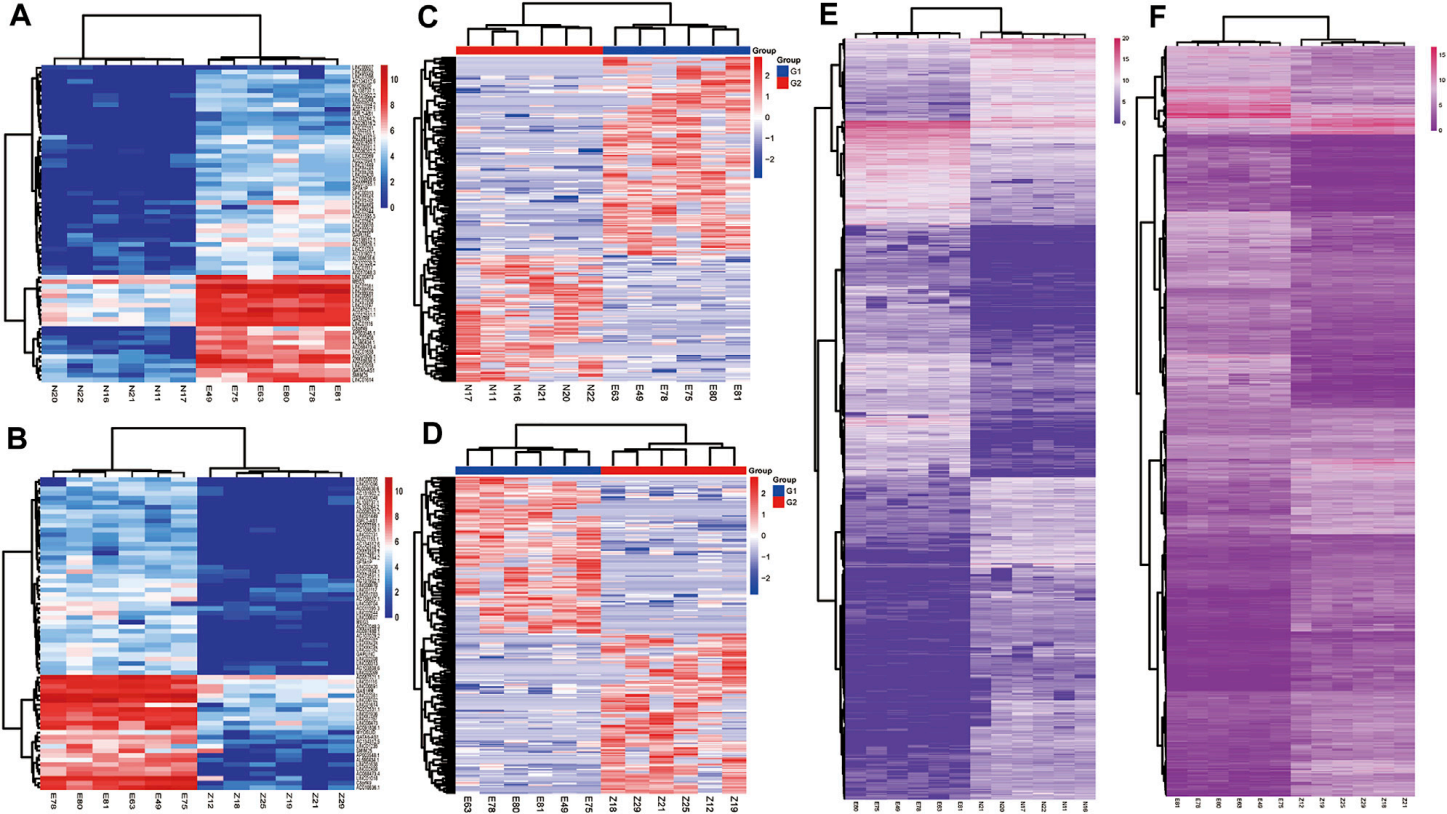
Heatmaps of DElncRNA, circRNA and mRNA in normal endometrium vs. ectopic endometrium and eutopic endometrium vs. ectopic endometrium datasets. **(A)** DElncRNA in normal endometrium vs. ectopic endometrium **(B)** DElncRNA in eutopic endometrium vs. ectopic endometrium datasets **(C)** DEcircRNA in normal endometrium vs. ectopic endometrium **(D)** DEcircRNA in eutopic endometrium vs. ectopic endometrium datasets. **(E)** DEmRNA in normal endometrium vs. ectopic endometrium **(F)** DEmRNA in eutopic endometrium vs. ectopic endometrium datasets.

According to the cut off criteria (FDR<0.0001 and |logFC|>3), a total of 140 DElncRNAs (68 up-regulated and 72 down-regulated) and 1,206 DEmRNAs(568 upregulated and 6,388 down regulated) were identified respectively. Owing to the different expression levels in the samples, differentially expressed circRNAs were screened according to |logFC| > 1, *p*＜0.05. A total of 107 circRNAs were differentially expressed in the two expression profiles(Figure 1). Heatmaps of DEcircRNA, DEmRNA and DElncRNA in their respective expression profiles are shown in Figure 2. In the following study, we mainly focused on the highly expressed DElncRNA and DEcircRNA.

### GSEA of DEmRNA

**FIGURE 3:**
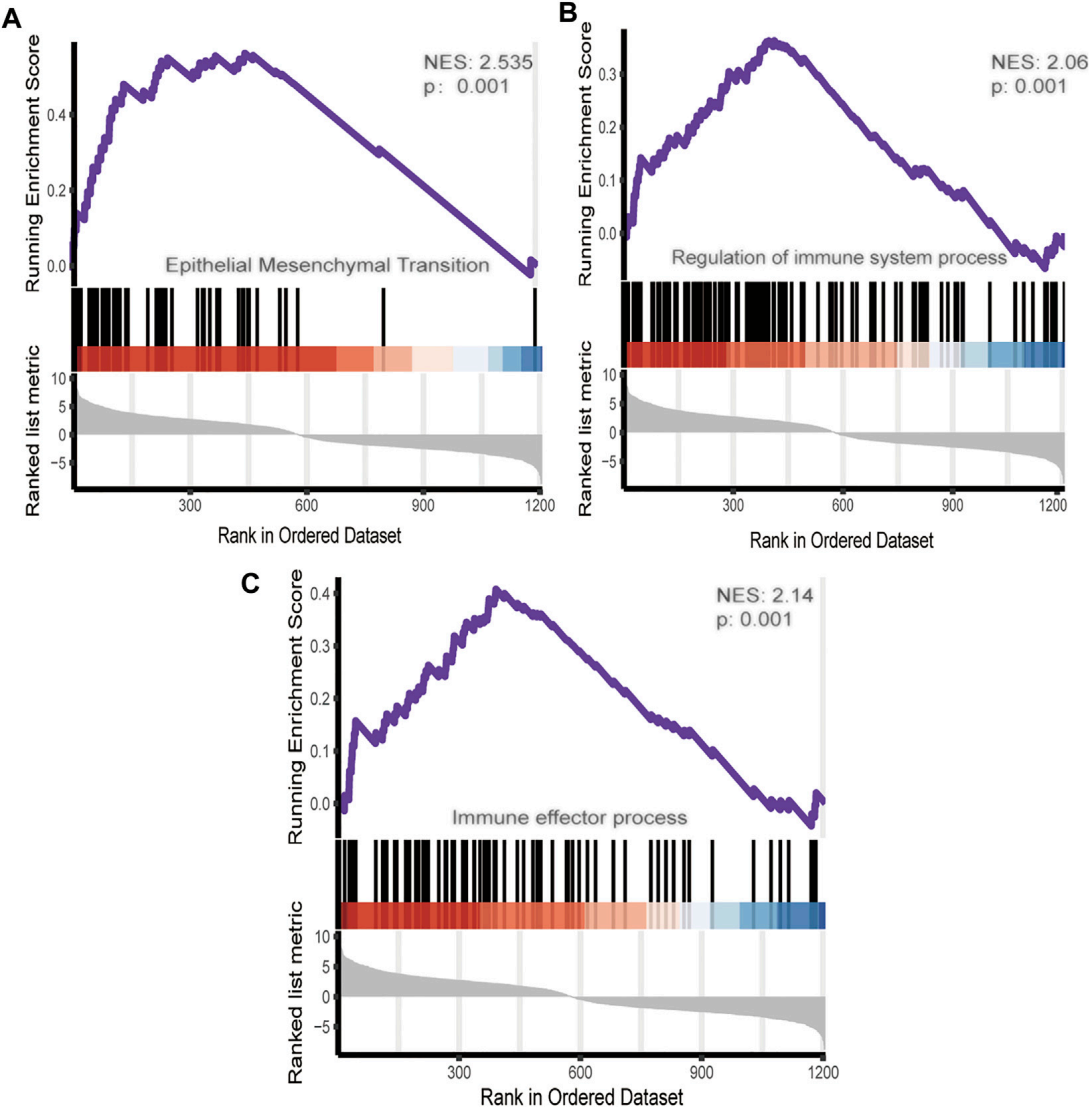
GSEA of DEmRNA.

To further investigate the role of DEmRNA expression in the endometriosis microenvironment. Gene set enrichment analysis was conducted by utilizing the gene expression profiles of 568 overexpressed DEmRNA (Supplementary Table). The gene signatures implied enrichment in many categories, such as epithelial-mesenchymal transition, regulation of immune system process and immune effector process(Figure 3).

### Circ/IncRNA-mRNA Co-Expression Network

**FIGURE 4:**
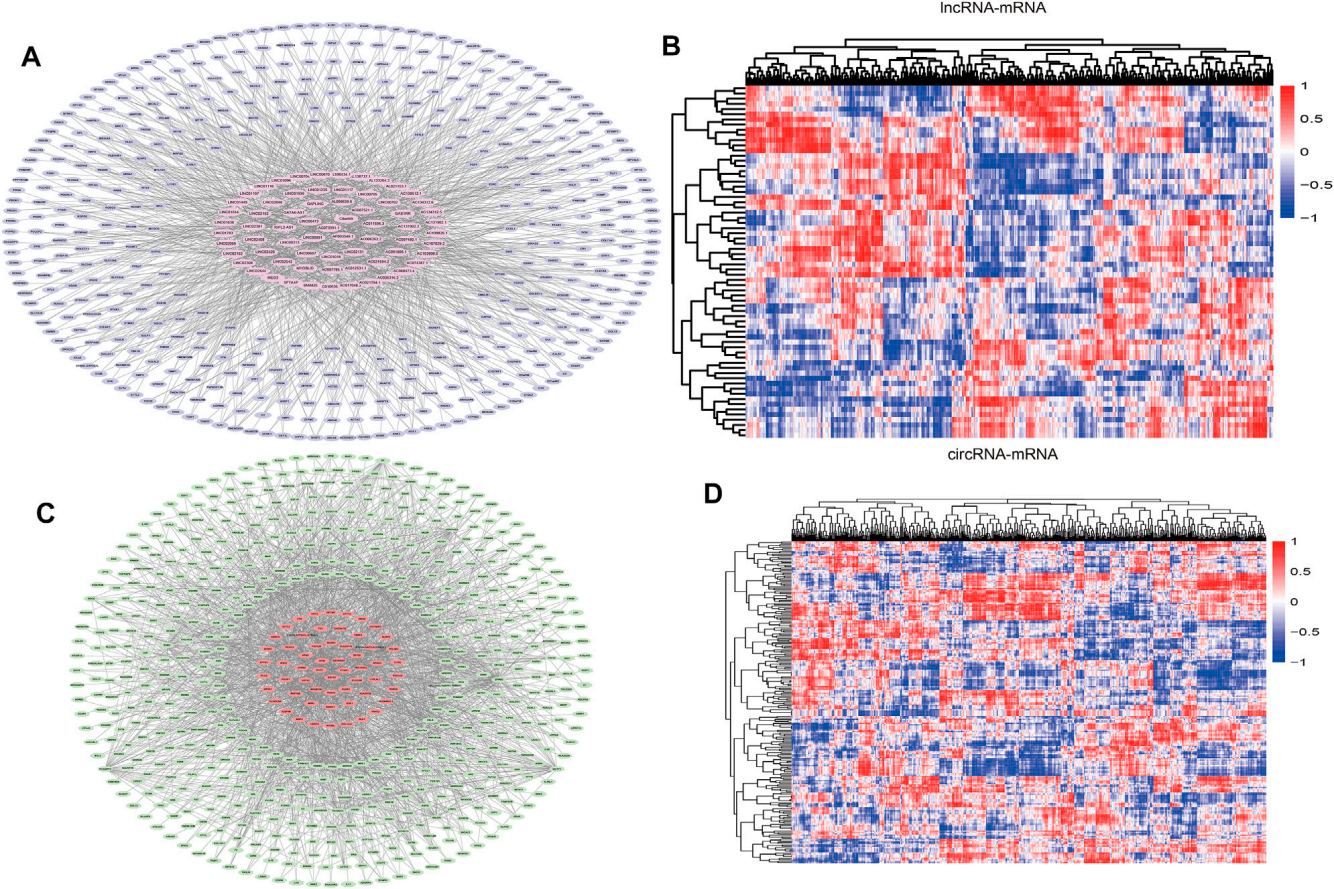
DElncRNA-DEmRNA pairs and DEcircRNA-DEmRNA pairs. **(A)** Networks of DElncRNA-DEmRNA, The pink hexagon in the middle represents DElncRNA, the outer purple oval represents DEmRNA. **(B)** Pearson correlation heatmaps of DElncRNA-DEmRNA. The color of each block represents the pearson correlation coefficient, red is positive, blue is negative. **(C)** Networks of DEcircRNA-DEmRNA. The red oval in the middle represents DEcircRNA, the outer green hexagon represents DEmRNA. **(D)** Pearson correlation heatmaps of DEcircRNA-DEmRNA.

Pearson correlation analysis was performed on DEcirc/DElncRNAs and DEmRNAs to select DEcirc/DElncRNA–DEmRNA pairs (*p* < 0.05 and rho ≥ 0.6). Meanwhile, by constructing pearson correlation analysis to establish ceRNA, circ/lnc/mRNA with unrelated expression were excluded. In total, 68 lncRNAs, 94 circRNA and 546 mRNAs were included in the co-expression network. circ/lncRNA-mRNA pairs are depicted via cytoscape software in Figures 4A,C, circRNA-mRNA network was composed of 627 nodes and 1817 edges. lncRNA-mRNA network was composed of 523 nodes and 1,203 edges. Correlation heatmaps of circRNA/lncRNA-mRNA are shown in Figures 4B,D.

### Prediction and Identification miRNAs Targeted by Both DElncRNA and DEcircRNA

**FIGURE 5:**
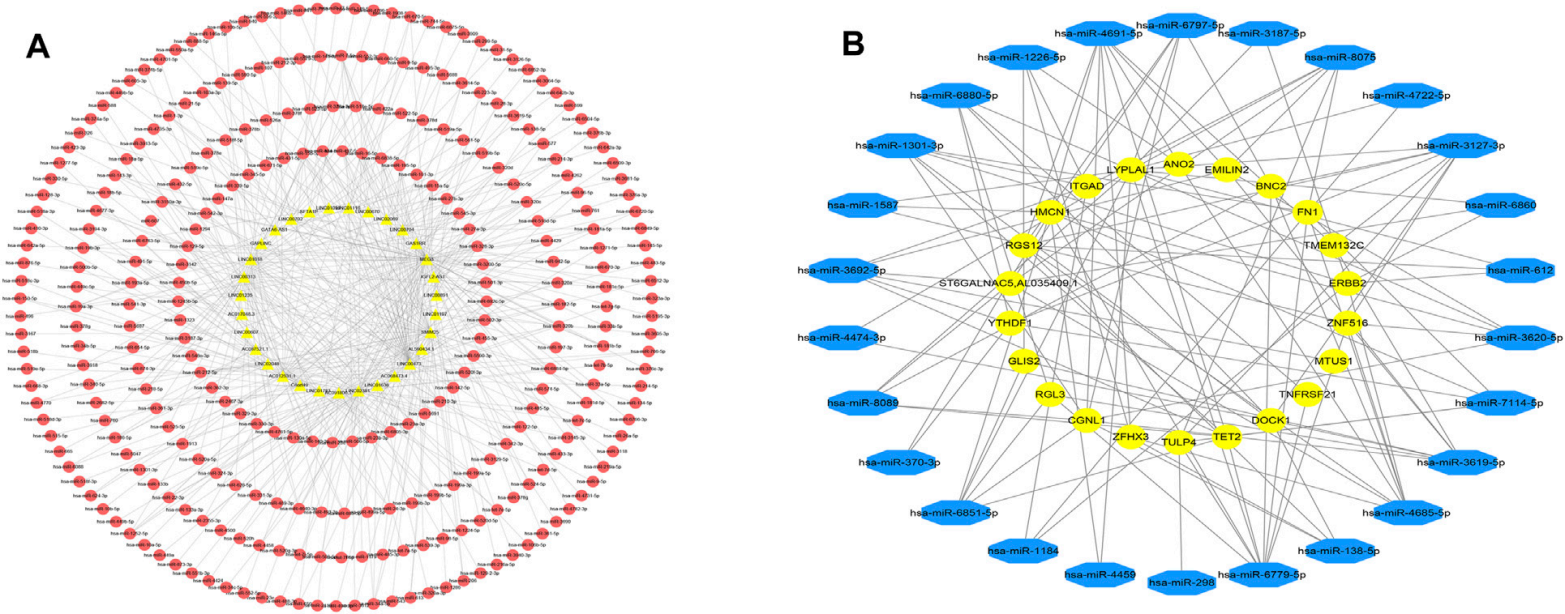
DElncRNA-miRNA and DEcircRNA-miRNA pairs. **(A)** Networks of DElncRNA-miRNA, yellow triangles represent lncRNAs and red circles represent miRNAs. **(B)** Networks of DEcircRNA-miRNA, yellow circle represents circRNA and the red octagon represents miRNA.

The starbase database was employed to predict the targeting miRNA of 68 DElncRNAs. Since lncRNAs and miRNAs are not one-to-one correspondences and some lncRNA do not predict miRNAs in the database, we obtained the 452 interaction between 31 lncRNAs and 294 miRNAs (Figure 5A).

There were 550 interacting circRNA-miRNA pairs predicted between 50 DECs and 50 miRNAs by the miRanda database in normal vs. ectopic datasets. Moreover there were 486 circRNA-miRNA pairs between 50 DECs and 50 miRNAs according to the miRanda database in eutopic vs. ectopic datasets. The circRNA-miRNA pairs mentioned above all met the conditions of S > 150, ΔG<−20 kcal/mol and Demand strict 5′ seed pairing. Among them, 120 pairs of circRNA-miRNAs were shared by the two data sets(Figure 5B).

**FIGURE 6:**
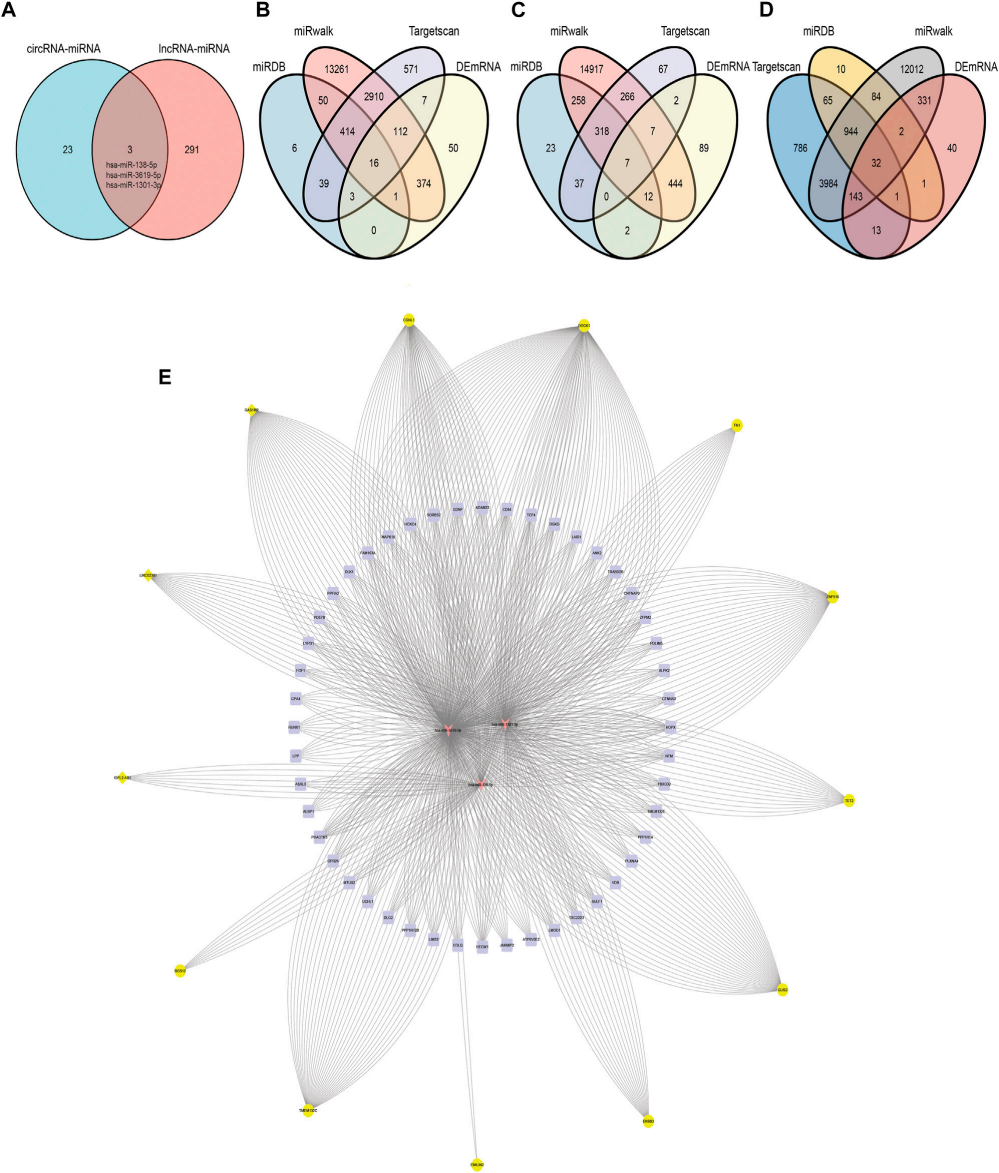
circRNA/lncRNA-miRNA-mRNA. **(A)** Common miRNA of circRNA-miRNA pairs and lncRNA-miRNA pairs. **(B)** Target mRNA of hsa-miR-1301-3p in miRDB, miRwalk, Targetscan and DEmRNA. **(C)** Target mRNA of hsa-miR-138-5p in miRDB, miRwalk, Targetscan and DEmRNA. **(D)** Target mRNA of hsa-miR-3619-5p in miRDB, miRwalk, Targetscan and DEmRNA. **(E)** Co-expression network of circRNA/lncRNA-miRNA-mRNA, 16 yellow circles in the outermost circle represent ncRNAs, the three orange arrows in the innermost circle represent miRNAs, and the 49 purple squares in the middle are mRNAs.

miRNAs shared by circRNA-miRNA and lncRNA-miRNA were selected as common miRNA in the co-expression network for further analysis. As we can seen in Figure 6A, hsa-miR-138-5p, hsa-miR-3619-5p and hsa-miR-1301-3p can bind to miRNA response elements of lncRNA and circRNA.

### Prediction of miRNA-mRNA Targeting Relationship

miRDB, TargetScan and miRwalk2.0 were simultaneously used to predict putative miRNA–mRNA interactions. In order to further improve the accuracy of prediction, we took the intersection of the predicted results of each miRNA in the three databases. Besides, the miRNA-mRNA pairs predicted by the three databases were not the final mRNAs that our co-expression network wanted to study. Only the predicted mRNAs presented in DEmRNA that we screened previously and related to circRNA/lncRNA expression were included in this co-expression network (Figures 6B–D). hsa-miR-1301-3p could bind to the 3 ′UTR of 16 DEmRNAs based on database prediction and expression correlation analysis. Similarly, hsa-miR-138-5p and hsa-miR-3619-5p could bind to 7 and 32 DEmRNAs respectively.

### Construction of ceRNA Network

**FIGURE 7:**
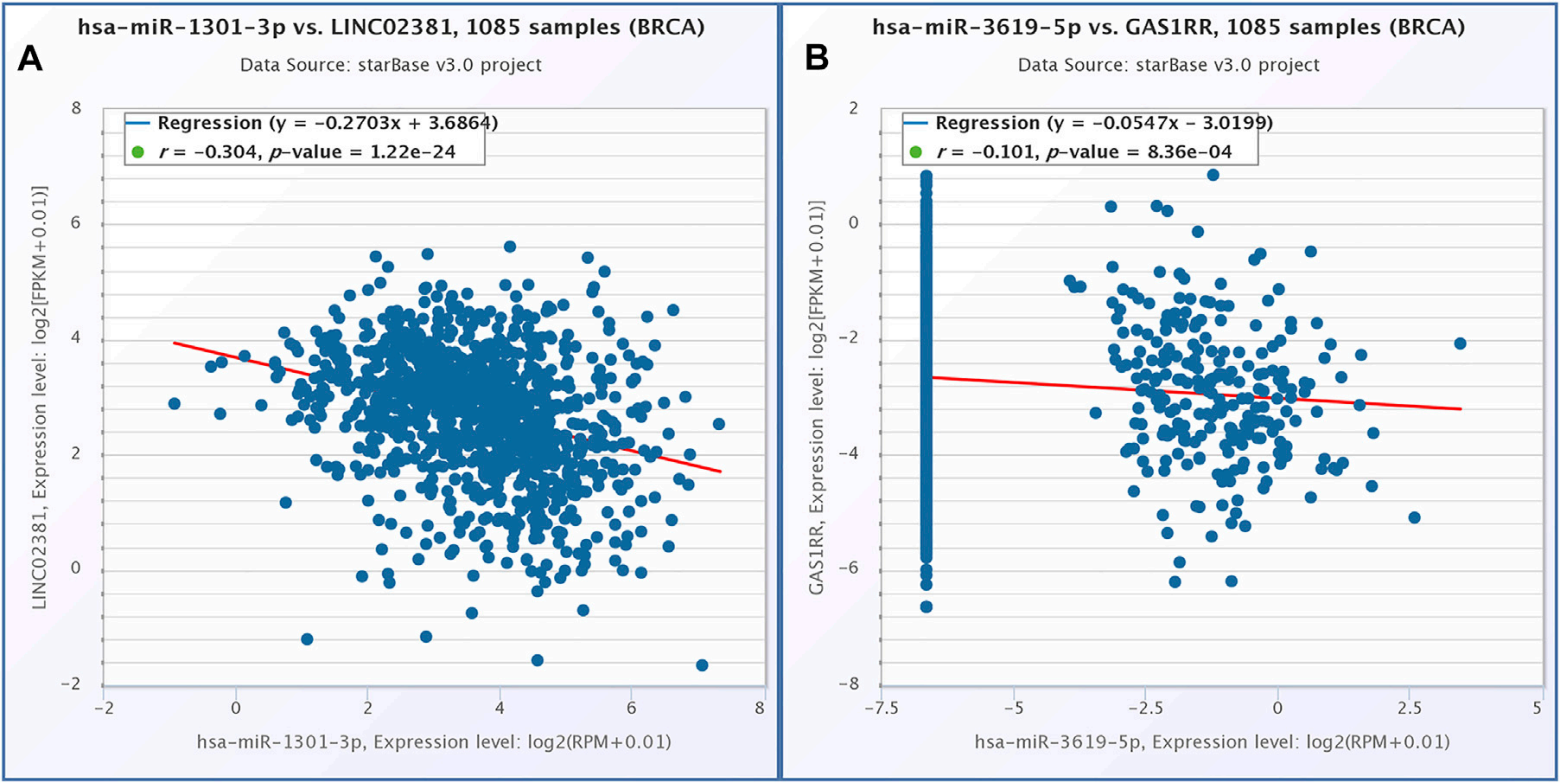
Correlation expression analysis of BRCA in starbase database. **(A)** LINC02381 and hsa-miR-1301-3p. **(B)** GAS1RR and hsa-miR-3619-5p.

According to the above analysis and prediction, there was a circRNA/lncRNA-miRNA-mRNA co-expression network among DEcircRNA, DElncRNA and DEmRNA in our sequencing results. The co-expression network consisted of three lncRNAs, 13 circRNAs, three miRNAs and 49 mRNAs. Among them, lncRNAs and mRNA were significantly differentially expressed in normal endometrium vs. ectopic endometrium and eutopic vs. ectopic endometrium (logFC＞3, FDR＜0.0001). circRNA met the condition of |logFC| > 1, *p* < 0.05 in the same two datasets as lncRNA and mRNA. The pearson correlation coefficient of circRNA/lncRNA-mRNA expression was greater than 0.6(*p*＜0.05) (Figure6E) According to starbase database, LINC02381-hsa-miR-1301-5p and GAS1RR-hsa-miR-3619-5p exhibited negative correlation in 1,085 breast invasive carcinoma samples. This is consistent with the ceRNA theory (Figure 7). S and ΔG of circRNA-miRNA pairs in co-expression network are shown in Table 2. The feasibility of this network was demonstrated from the perspective of expressing relevance and database prediction. However, further experimental verification is needed.

**TABLE 2:** Single-legs-pair match scores and free energy of double strand formation of circRNA-miRNA pairs in circRNA/lncRNA-miRNA-mRNA network.

circRNA	miRNA	Tot score	Tot energy
RGS12	hsa-miR-138-5p	164	−23.59
ZFHX3	hsa-miR-138-5p	317	−48.12
CGNL1	hsa-miR-138-5p	161	−21.78
DOCK1	hsa-miR-138-5p	156	−24.32
FN1	hsa-miR-1301-3p	157	−22.74
ITGAD	hsa-miR-1301-3p	163	−26.58
DOCK1	hsa-miR-1301-3p	155	−20.91
TET2	hsa-miR-1301-3p	161	−25.18
ERBB2	hsa-miR-1301-3p	150	−25.62
CGNL1	hsa-miR-1301-3p	153	−22.39
CGNL1	hsa-miR-3619-5p	155	−21.89
ZNF516	hsa-miR-3619-5p	306	−48.61
GLIS2	hsa-miR-3619-5p	320	−50.62
DOCK1	hsa-miR-3619-5p	317	−52.08
EMILIN2	hsa-miR-3619-5p	153	−24.32
RGL3	hsa-miR-3619-5p	174	−37.37
TMEM132C	hsa-miR-3619-5p	152	−22.05

### Reverse Transcription Quantitative PCR

**FIGURE 8:**
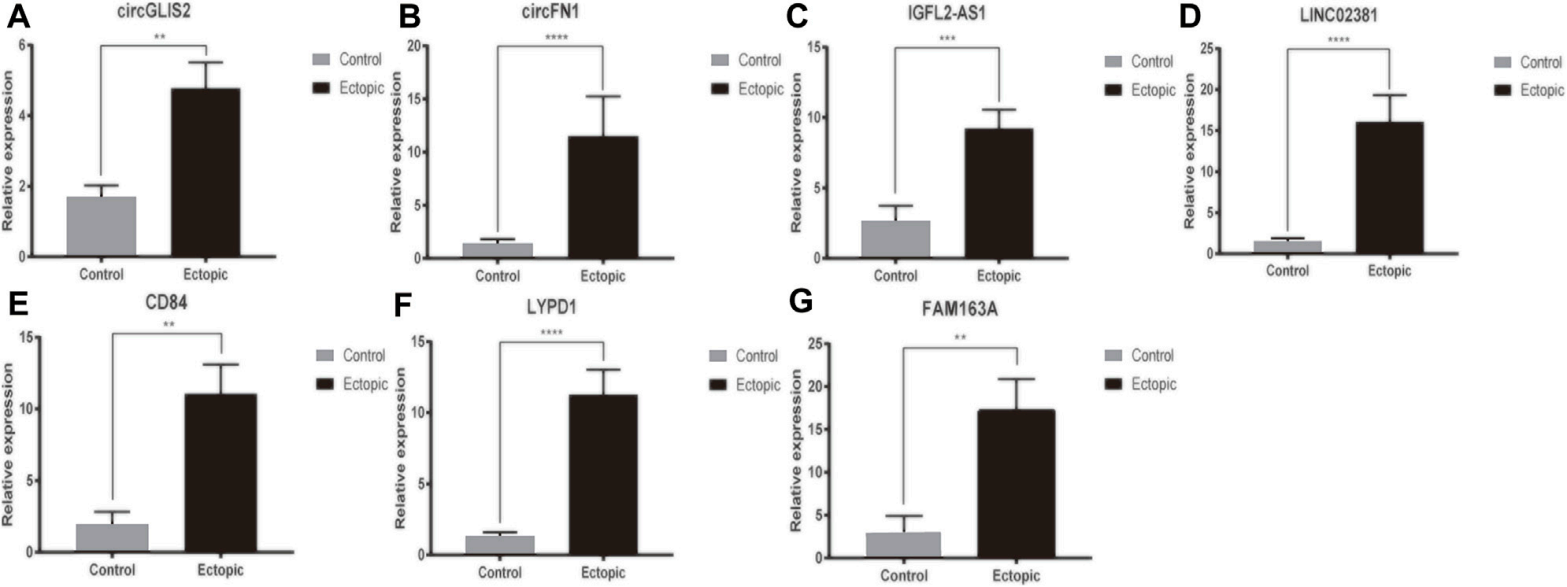
RT-qPCR of DEcircRNA, DElncRNA and DEmRNA in co-expression network.

As mentioned above, circRNA, lncRNA and mRNA in the co-expression network were significantly differentially expressed in ectopic endometrial samples. RT-qPCR was utilized to detect the expression of DERNAs in 25 ectopic endometrial tissues and 15 negative control endometrial tissues(Figure 8). We focused on the two up-regulated lncRNA (IGFL2-AS1, LINC02381) and two up-regulated circRNA (circGLIS2, circFN1) for further research. Among the mRNA in the ceRNA network composed of LINC02381-hsa-miR-1301-3p pairs in co-expression networks, the mRNA with the highest pearson correlation coefficient with LINC02381 was selected for RT-qPCR validation (LYPD1). The mRNA with the strongest correlation with circGLIS2 and circFN1 was also found by the same method (CD84, FAM163A) Compared with the control group, the ncRNA, including circGLIS2, circFN1, LINC02381, IGFL2-AS1, were significantly overexpressed and consistent with the RNA sequencing results. CD84, LYPD1, and FAM163A were also significantly overexpressed in ectopic tissues. Spearman correlation analysis based on our 40 samples indicated that LINC02381 was positively associated with LYPD1 (*r* = 0.592, *p* = 0.000057), circFN1 was positively associated with CD84 (*r* = 0.533, *p* = 0.001), and circGLIS2 was positively associated with FAM163A (*r* = 0.572, *p* = 0.000114). These results were consistent with our above analysis above.

## Discussion

Using the forefront technology in microarray analysis, we demonstrated the expression profiles of human lncRNAs, circRNAs and mRNAs in patients with EMS for the first time. Compared to matched controls, these EMS patients expressed 140 lncRNAs, 107 circRNAs and 1,206 mRNAs that did not appear in the control group. Moreover, we identified potential functions of these differentially expressed mRNAs with GSEA and established circRNA/lncRNA-miRNA-mRNA in EMS.

The results of this study showed that the expressions of four ncRNAs (circGLIS2, circFN1, IGFL2-AS1 and LINC02381) and three mRNA (CD84, LYPD1, FAM163A) were markedly different between EMS tissues and control endometrial tissues. Many of these are still incompletely studied.

circFN1-CD84 pair was confirmed to be highly expressed in EMS patients in our analysis. circFN1 has been shown in previous studies to be related to the occurrence of drug resistance to sorafenib in hepatocellular carcinoma and cisplatin in gastric cancer via ceRNA (Chen C et al., 2020; Huang et al., 2020). CD84 (SLAMF5) is a member of the signaling lymphocyte activation molecule (SLAM) family, which are cell surface proteins committed to regulating immune response (Silvia et al., 2008). CD84 mediated signaling regulates a variety of immune processes, including natural killer cytotoxicity, T cytokine secretion, monocyte activation, autophagy, homologous T, B interaction and B cell tolerance at germinal center checkpoints (Marta et al., 2019). Recently, endometriosis has been considered as an autoimmune disease (AID) in view of the presence of autoantibodies, high cytokine levels, therapeutic sensitivity to immunomodulators and co-currence with other AID (Hila et al., 2021). Studies in the 1980s found that the dysregulation of host immune system is the main process leading to endometriosis. Since then, changes in the immune system including cell-mediated immunity and humoral immunity in endometriosis have been identified (Symons et al., 2018). B lymphocyte activation in EMS may result in the formation of a variety of autoantibodies The deposition of immunoglobulin and complement in endometrium indicates the role of immune complex formation in the pathogenesis of EMS. In summary, a disordered immune response plays an integral role in the pathogenesis of EMS. Consistent with the previous research results, we found that circFN1 affected the expression of CD84 by sponging hsa-miR-1301-3p in EMS, thus affecting the immune related activities of EMS.

Bioinformatics analyses indicated that LINC02381 as a ceRNA can sponge hsa-miR-1301-3p to modulate expression level of LYPD1. Several functional experiments have shown that LINC02381 can affect molecular pathogenesis of wide a variety of diseases as ceRNA. In rheumatoid arthritis, upregulation of LINC02381 can complement with miR-590-5p to reduce its level in RA tissues and inhibit the expression of mitogen-activated protein kinase 3 (MAP2K3) at the post-transcription level (Sun et al., 2021). In colorectal carcinoma (CRC), LINC02381 exhibit down-regulated expression in CRC tissues and different cell lines. This differential expression affects the growth and apoptosis of CRC cells through PI3K-Akt signaling pathway (Meisam et al., 2008). In gastric cancer, downregulated LINC02381 resulted in the increase of free miR-21, miR-590 and miR-27a, enhanced cell proliferation and ascension of EMT related markers. However, LINC02381 has never been reported in EMS. In our analysis, the 3’ UTR of CD84 and LYPD1 could combine with hsa-miR-1301-3p. This indicatesd that the expression of CD84 and LYPD1 can be regulated by LINC02381 (Wang and Zhao, 2020). For LYPD1, it is reported that LY6/PLAUR Domain containing 1 (LYPD1) is a novel therapeutic antibody target for ovarian cancer. LYPD1 is extensively expressed in both primary and metastatic ovarian cancer. Anti-LYPD1/CD3 T-cell-dependent bispecific antibody (TDB) can lead polyclonal T cells to activate and target ovarian cancer cells with LYPD1 expression (Amy et al., 2020). Moreover, LYPD1 has been recognized as an oncogenic driver in hepatocellular carcinoma (Chen J et al., 2020). LYPD1 can directly inhibit the formation of endothelial cell network, and regulate anti-angiogenic properties of cardiac fibroblasts. Interestingly, both circFN1 and LINC02381 can bind to hsa-miR-1301-3p, which can integrate with both CD84 and LYPD1. All these ncRNA and mRNA have been shown to be overexpressed in EMS. Therefore, according to this, we can infer that circFN1 and LINC02381 can trigger immune response dysregulation of EMS via manipulating expression of CD84 and LYPD1. Nevertheless, further experimental validation is urgently needed.

circGLIS2 is situated on the plus strand of chromosome 16p13.3 and derived from the known protein coding gene GLIS2. Chen et al. found that circGLIS2 was differentially expressed in colorectal cancer and it can activate NF-κB pathway by sponge miR-671 and further promote the production of pro-inflammatory chemokine (Chen Y et al., 2020). Except for this, circGLIS2 is poorly understood in other diseases. In our research, we demonstrated that circGLIS2 was differentially expressed in EMS tissues. Similarly, circGLIS2 can also regulate the occurrence and development of EMS diseases through ceRNA mechanism. circGLIS2 can also regulate the expression of FAM163A and binding with hsa-miR-3619-5p.

Since miRNA and mRNA combinations are not one-to-one correspondence, a single miRNA may bind to multiple mRNAs. FAM163A, located on chromosome 1q25.2 and encoding a 167-amino acid protein, is also recognized as neuroblastoma-derived secretory protein (NDSP) or C1ORF76. In squamous cell lung carcinoma, Liu et al. found that FAM163A interacts with 14-3-3β to promote ERK phosphorylation and thus affect lung cancer cell proliferation (Liu et al., 2019). This is the first time that up-regulated expression of FAM163A in EMS tissues has been shown under the regulation of circGLIS2. After reviewing previous studies, we speculated that FAM163A can also promote the proliferation of EMS cells, but further experiments are needed to demonstrate this.

lncRNA IGFL2-AS1 is an antisense transcript of IGF like family member 2 (IGFL2) gene. It is located on chromosome 19 with three exons. Accumulating evidence suggests that its aberrant expression is associated with regulation of cellular and pathological processes of several cancer through ceRNA, including gastric cancer (Ma et al., 2020), basal-like breast cancer (Wang et al., 2021). In EMS, we utilize next-generation sequencing to reveal differential expression of lncRNA IGFL2-AS1 and determined the ceRNA mechanism can combine with hsa-miR-138-5p to promote NTM expression. NTM, one of the target gene of lncRNA IGFL2-AS1, was shown in our GSEA analysis to participate in the epithelial-mesenchymal transition of EMS (Supplementary Material) Increasing numbers of experiments have improved that EMT is involved in inducing the invasion and migration of endometrial epithelial cells, and this process is of great significance in establishment of endometriosis (Matsuzaki and Darcha, 2012). Epithelial-mesenchymal transition (EMT) is the loss of polarity and change of epithelial cells to a mesenchymal phenotype. Epithelial cell invasiveness was increased during EMT. Endometriosis tissues were also shown to have more e-cadherin negative cells than healthy endometrium, while N-cadherin, Twist, Slug, and Snail were all elevated in endometriosis tissues (Bartley et al., 2014).

In summary, this work provided, for the first time, a preliminary overview of differentially expressed lncRNA, circRNA, and mRNA in EMS. This research adds to our understanding of the pathophysiology of EMS and offers clinical therapeutic options. However, *in vivo* and *in vitro* investigations are needed to confirm the precise function of lncRNA and circRNA in EMS.

## Data Availability

All datasets generated for this study are included in the article/Supplementary Material. For further details, please contact the corresponding author.
